# VERDICT MRI for Prostate Cancer: Intracellular Volume Fraction versus Apparent Diffusion Coefficient

**DOI:** 10.1148/radiol.2019181749

**Published:** 2019-04-02

**Authors:** Edward W. Johnston, Elisenda Bonet-Carne, Uran Ferizi, Ben Yvernault, Hayley Pye, Dominic Patel, Joey Clemente, Wivijin Piga, Susan Heavey, Harbir S. Sidhu, Francesco Giganti, James O’Callaghan, Mrishta Brizmohun Appayya, Alistair Grey, Alexandra Saborowska, Sebastien Ourselin, David Hawkes, Caroline M. Moore, Mark Emberton, Hashim U. Ahmed, Hayley Whitaker, Manuel Rodriguez-Justo, Alexander Freeman, David Atkinson, Daniel Alexander, Eleftheria Panagiotaki, Shonit Punwani

**Affiliations:** From the UCL Centre for Medical Imaging, University College London, 2nd Floor Charles Bell House, 43-45 Foley Street, London W1W 7TS, England (E.W.J., E.B.C., H.S.S., J.O., M.B.A., D. Atkinson, S.P.); UCL Centre for Medical Image Computing, London, England (E.B.C., U.F., B.Y., S.O., D.H., D. Alexander, E.P.); UCL Centre for Molecular Intervention, London, England (H.P., S.H., H.W.); Department of Histopathology, University College Hospital, London, England (D.P., M.R.J., A.F.); Department of Radiology (J.C.) and Centre for Medical Imaging (J.C., W.P., A.S.), University College Hospital, London, England; Division of Surgery and Interventional Science, Faculty of Medical Sciences, University College London, London, England (F.G., A.G., C.M.M., M.E.); and Department of Surgery and Cancer, Imperial College London, London, England (H.U.A.).

## Abstract

**Background:**

Biologic specificity of diffusion MRI in relation to prostate cancer aggressiveness may improve by examining separate components of the diffusion MRI signal. The Vascular, Extracellular, and Restricted Diffusion for Cytometry in Tumors (VERDICT) model estimates three distinct signal components and associates them to *(a)* intracellular water, *(b)* water in the extracellular extravascular space, and *(c)* water in the microvasculature.

**Purpose:**

To evaluate the repeatability, image quality, and diagnostic utility of intracellular volume fraction (FIC) maps obtained with VERDICT prostate MRI and to compare those maps with apparent diffusion coefficient (ADC) maps for Gleason grade differentiation.

**Materials and Methods:**

Seventy men (median age, 62.2 years; range, 49.5–82.0 years) suspected of having prostate cancer or undergoing active surveillance were recruited to a prospective study between April 2016 and October 2017. All men underwent multiparametric prostate and VERDICT MRI. Forty-two of the 70 men (median age, 67.7 years; range, 50.0–82.0 years) underwent two VERDICT MRI acquisitions to assess repeatability of FIC measurements obtained with VERDICT MRI. Repeatability was measured with use of intraclass correlation coefficients (ICCs). The image quality of FIC and ADC maps was independently evaluated by two board-certified radiologists. Forty-two men (median age, 64.8 years; range, 49.5–79.6 years) underwent targeted biopsy, which enabled comparison of FIC and ADC metrics in the differentiation between Gleason grades.

**Results:**

VERDICT MRI FIC demonstrated ICCs of 0.87–0.95. There was no significant difference between image quality of ADC and FIC maps (score, 3.1 vs 3.3, respectively; *P* = .90). FIC was higher in lesions with a Gleason grade of at least 3+4 compared with benign and/or Gleason grade 3+3 lesions (mean, 0.49 ± 0.17 vs 0.31 ± 0.12, respectively; *P* = .002). The difference in ADC between these groups did not reach statistical significance (mean, 1.42 vs 1.16 × 10^-3^ mm^2^/sec; *P* = .26).

**Conclusion:**

Fractional intracellular volume demonstrates high repeatability and image quality and enables better differentiation of a Gleason 4 component cancer from benign and/or Gleason 3+3 histology than apparent diffusion coefficient.

Published under a CC BY 4.0 license.

*Online supplemental material is available for this article.*

See also the editorial by Sigmund and Rosenkrantz in this issue.

**Summary**

The intracellular volume fraction derived from Vascular, Extracellular, and Restricted Diffusion for Cytometry in Tumors (VERDICT) MRI enables better differentiation of a Gleason 4 lesion from benign and/or Gleason grade 3+3 lesions in prostate cancer with a high level of repeatability and similar image quality compared with apparent diffusion coefficient values.

**Key Points**

■ The repeatability of the Vascular, Extracellular, and Restricted Diffusion for Cytometry in Tumor (VERDICT) MRI model was high (intraclass correlation coefficient, 0.87–0.95)
■ The intracellular volume fraction in prostate lesions with a Gleason grade of at least 3+4 was higher than that in benign lesions and/or those with a Gleason grade of 3+3 (mean, 0.49 vs 0.31, respectively; *P* = .002).
■ The apparent diffusion coefficients for lesions with a Gleason grade of at least 3+4 were not significantly different from those of benign lesions and/or those with a Gleason grade of 3+3 **(**mean, 1.42 vs 1.16 × 10^-3^ mm^2^/sec; *P* = .26).

## Introduction

Despite the merits of the apparent diffusion coefficient (ADC), reporting quantitative ADC values is not a routine part of clinical practice. This is partially due to lack of biologic specificity (1). Recently, our group presented the feasibility of Vascular, Extracellular, and Restricted Diffusion for Cytometry in Tumors (VERDICT) MRI as a quantitative microstructural imaging tool for prostate cancer (2). VERDICT combines a diffusion-weighted MRI acquisition with a mathematical model and assigns the diffusion-weighted MRI signal to three principal components: *(a)* intracellular water, *(b)* water in the extracellular extravascular space, and *(c)* water in the microvasculature. Because the fraction of each of these compartments differs between each Gleason grade (3), we hypothesized that VERDICT-derived metrics may provide higher biologic specificity than ADC as a marker of prostate cancer aggressiveness.

We performed this study to evaluate the repeatability, image quality, and diagnostic utility of intracellular volume fraction (FIC) maps obtained with VERDICT prostate MRI and to compare those maps with ADC maps for Gleason grade differentiation.

## Materials and Methods

Ethical approval for the study was granted by the London–Surrey Borders Research Ethics Committee. The trial is registered with ClinicalTrials.gov identifier NCT02689271. Our institutional review board approved the study protocol, and written informed consent was obtained from all study participants.

Our study was carried out as part of a prospective cohort study. The full study protocol has been published previously (4). Prostate Cancer UK funded the study.

### Study Participants

Potentially eligible participants were identified at University College Hospital from a list of men scheduled to undergo conventional multiparametric MRI. Men meeting the eligibility criteria were approached to form a consecutive series. Men were included if *(a)* there was clinical suspicion of prostate cancer or *(b)* they were undergoing active surveillance for known prostate cancer. Men were excluded if *(a)* they had previously undergone treatment for prostate cancer (prostatectomy, radiation therapy, brachytherapy, ablative therapies), *(b)* they were undergoing ongoing hormonal treatment for prostate cancer, and *(c)* they had undergone biopsy within 6 months before multiparametric MRI (4). In total, 72 men were recruited for VERDICT MRI between April 2016 and October 2017.

**Figure 1:**
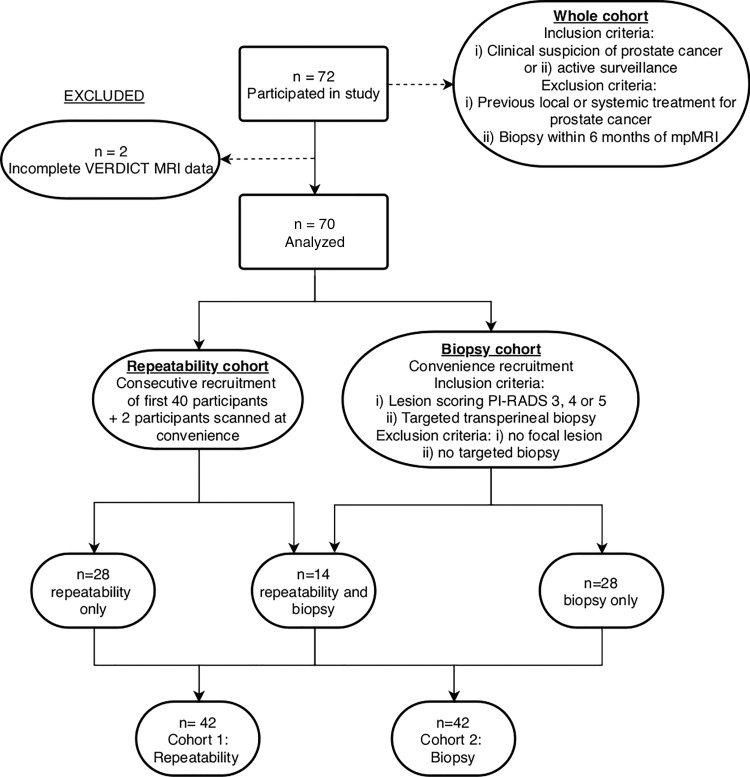
Flow diagram of participant recruitment. mpMRI = multiparametric MRI, PI-RADS = Prostate Imaging Reporting and Data System, VERDICT = Vascular, Extracellular and Restricted Diffusion for Cytometry in Tumors.

Two study participants were excluded due to incomplete VERDICT MRI data sets. Thus, imaging data from 70 participants (median age, 62.2 years; range, 49.5–82.0 years) were used to form two cohorts: cohort 1, the repeatability cohort, and cohort 2, the biopsy cohort. A participant recruitment flow diagram is presented in Figure 1.

To evaluate repeatability of VERDICT MRI metrics, a scan-rescan repeatability study of the VERDICT MRI acquisition protocol was performed in 42 participants (median age, 67.7 years; range, 50.0–82.0 years). Here, participants were imaged twice, with less than 5 minutes between each examination.

The biopsy study was performed to compare FIC and ADC metrics for the differentiation between Gleason grades. After clinical multiparametric MRI and VERDICT MRI, 42 participants (median age, 64.8 years; range, 49.5–79.6 years) underwent targeted transperineal template biopsy of their index lesion. Multiparametric MRI was used to guide cognitive targeted biopsy (performed by urologists H.U.A. and C.M.M., each with 7 years of targeted biopsy experience). Fourteen of the 42 patients were also included in the repeatability cohort. Specialist genitourinary pathologists (including A.F. and M.R., with 13 and 15 years of prostate pathology experience, respectively) evaluated histologic examinations from the biopsy cores in the standard clinical fashion and assigned each biopsy core a Gleason grade (5). Because there is a clinical need to differentiate tumors with a Gleason 4 component, we grouped results into three categories: benign and/or Gleason grade 3+3 lesions, Gleason grade 3+4 lesions, and lesions with a Gleason grade of at least 4+3.

### Image Acquisition

***ADC.—***All participants underwent multiparametric MRI with a 3.0-T MRI system (Achieva; Philips, Best, the Netherlands) as part of their standard clinical care. A spasmolytic agent (Buscopan, Boehringer Ingelheim, Ingelheim am Rhein, Germany; 0.2 mg/kg, up to 20 mg) was administered intravenously before imaging to reduce bowel peristalsis. Imaging parameters for the diffusion-weighted echo-planar imaging sequences that generated the ADC map were as follows: repetition time msec/echo time msec, 2753/80; field of view, 220 × 220 mm; section thickness, 5 mm; no intersection gap; acquisition matrix, 168 × 169 mm; *b* values, 0, 150, 500, and 1000 sec/mm^2^; and six signals acquired per *b* value. The total imaging time for the clinical diffusion-weighted sequences was 5 minutes 16 seconds. ADC maps were calculated by using all *b* values except *b* = 0 to reduce perfusion effects (6) and were calculated with the Camino Diffusion MRI toolkit (7).

Full acquisition parameters for multiparametric MRI are provided in Table E1 (online).

***VERDICT MRI.—***VERDICT MRI was performed before dynamic contrast material–enhanced imaging on the same 3.0-T unit as the clinical multiparametric MRI acquisition. Sequences used an echo-planar readout, and imaging parameters were as follows: 2482–3945/50–90; field of view, 220 × 220 mm; section thickness, 5 mm; no intersection gap; acquisition matrix, 176 × 176 mm; *b* values, 90, 500, 1500, 2000, and 3000 sec/mm^2^; and six signals acquired per *b* value (except for *b* = 90 sec/mm^2^, which used four signals acquired). The total imaging time was 12 minutes 25 seconds.

VERDICT MRI parameters are provided in Table E2 (online), and further details regarding the biophysical basis and optimization of VERDICT have been previously described by Panagiotaki et al (2,8).

**Figure 2:**
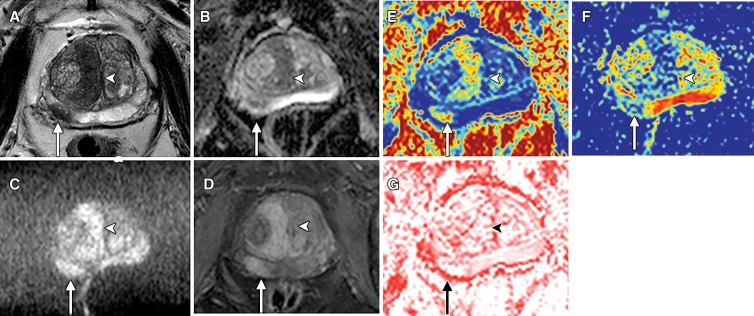
Images in an 82-year-old man with biopsy-proven prostate cancer arising in both the transition zone (arrows) and peripheral zone (arrowheads). *A*, Axial T2-weighted turbo spin-echo MRI shows lenticular right paramidline transition zone tumor and right peripheral zone tumor at 7 to 8 o’clock. Both have low signal intensity. *B*, Apparent diffusion coefficient (ADC) map shows that both tumors have reduced ADC. *C*, MRI obtained with *b* value of 2000 sec/mm^2^ shows tumors with high signal intensity. *D*, Early dynamic MRI obtained with gadolinium-based contrast material shows enhancement of both lesions. *E*, Axial Vascular, Extracellular, and Restricted Diffusion for Cytometry in Tumors (VERDICT) map of intracellular volume fraction shows tumors with increased intracellular volume fraction. *F*, VERDICT map of extracellular extravascular volume fraction shows reduction in degree of extracellular extravascular space. *G*, VERDICT map of vascular volume fraction shows tumors with equivocal-to-low vascular volume fraction values. Methods for determining extracellular extravascular volume fraction and vascular volume fraction, along with the results, are provided in Appendix E1 (online).

VERDICT MRI maps (as shown in Fig 2) were generated by using the accelerated microstructure imaging via convex optimization, or AMICO, framework, which has previously been described by Bonet-Carne et al (9). The methods used for and results of extravascular extracellular volume fraction and vascular volume fraction analyses are presented in Appendix E1 (online).

### Image Analysis

***Multiparametric MRI lesion localization.—***Multiparametric MRI studies were evaluated by a uroradiologist (S.P., with 10 years of prostate multiparametric MRI reporting experience) and scored by using Prostate Imaging Reporting and Data System (PI-RADS) version 2 (10). Where multiple lesions were present, the most conspicuous lesion with the highest PI-RADS score (3, 4, or 5) was defined as the index lesion.

***Quantitative assessment of FIC and ADC.—***FIC and ADC maps were analyzed with software (Osirix, version 8.0; Osirix, Pixmeo SARL, Bernex, Switzerland). A board-certified radiologist (E.W.J., with 3 years of experience in multiparametric MRI) manually drew a region of interest (ROI) for each index lesion on each map. Where possible, additional ROIs were placed in PI-RADS category 1–2 lesions in the transition zone and peripheral zone. For evaluation of FIC repeatability, ROIs were copied onto maps generated from the second VERDICT MRI acquisition. Mean FIC and ADC values from the ROIs were recorded.

***Assessment of FIC and ADC map image quality.—***Two board-certified radiologists (F.G. and H.S.S., fellows in prostate MRI with 5 years of experience in multiparametric MRI), who were unaware of the study purpose, independently assessed anonymized randomized ADC and FIC maps (displayed in gray scale). Overall image quality was scored by using a subjective five-point ordinal scale in accordance with that reported by Heijmink et al (11) and Barth et al (12). A full definition of the image quality scale is provided in Table E3 (online).

For the biopsy cohort, quantitative measurements of contrast-to-noise ratio were calculated for each index lesion according to the study by Grussu et al (13).

### Gleason Grade Differentiation with FIC and ADC

Mean quantitative ROI ADC and FIC metrics were paired with location-matched biopsy results as reported by the pathologists. ADC and FIC values measured for each focal prostate lesion were assigned to different histopathologic categories (benign, Gleason grade of 3+3, Gleason grade of 3+4, and Gleason grade 4+3).

### Statistical Analysis

Data were analyzed with software (SPSS, version 22 [IBM, Armonk, NY] and GraphPad Prism 6.0e [GraphPad, La Jolla, Calif]).

Normality was checked by using the Shapiro-Wilk test. FIC repeatability was assessed with intraclass correlation coefficients (ICCs) (1,3) and Bland-Altman analysis as recommended by Sullivan et al (14).

FIC and ADC map image quality scores were compared (together with extravascular extracellular volume fraction and vascular volume fraction, Appendix E1 [online]) by using the Friedman test with Dunn’s multiple comparison correction. The weighted kappa (κ) was calculated to assess agreement between the two readers for the overall image quality review.

Analysis of variance with Bonferroni multiple comparisons correction was performed to determine the differences between three defined histopathologic categories (benign and/or Gleason grade of 3+3, Gleason grade of 3+4, and Gleason grade ≥4+3) for ADC and FIC. FIC and ADC receiver operating characteristic curves were plotted to differentiate benign and/or Gleason 3+3 lesions from lesions with a Gleason grade of at least 3+4, and the area under the receiver operating characteristic curve was recorded.

## Results

There were 61 index lesions in the 70 participants. Nine participants had no focal lesion on multiparametric MRI (PI-RADS category 2), 28 had a PI-RADS category 3 lesion, 19 a PI-RADS category 4 lesion, and 14 a PI-RADS category 5 lesion. Median prostate-specific antigen level was 7.0 ng/mL (range, 1.0–71.0 ng/mL).

For the biopsy cohort (*n* = 42), the median time between VERDICT MRI and biopsy was 66.9 days (range, 8–167 days). Of the 42 biopsied index lesions, 15 were benign, five were Gleason grade 3+3, 11 were Gleason grade 3+4, and 11 were Gleason grade 4+3 or greater. A summary of the demographic data is provided in Table 1.

**Table:**
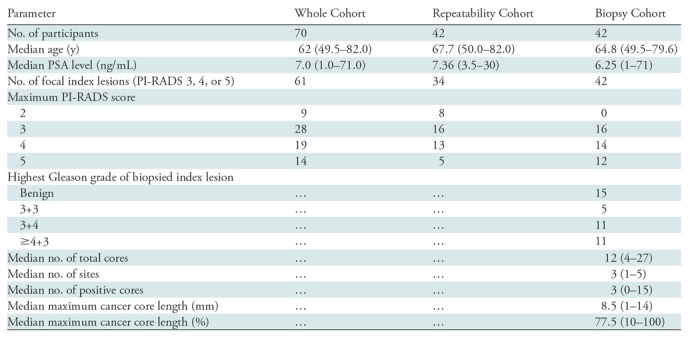
Summary of Demographic Data

Note.—Except where indicated, data are numbers of participants. Numbers in parentheses are ranges. PI-RADS = Prostate Imaging Reporting and Data System, PSA = prostate specific antigen.

### Metric Repeatability

The ICC for FIC (first VERDICT MRI vs second VERDICT MRI) in PI-RADS category 1 and 2 lesions was 0.88 (95% confidence interval: 0.77, 0.94) in the transition zone and 0.95 (95% confidence interval: 0.91, 0.98) in the peripheral zone. The ICC for FIC in PI-RADS category 3, 4, or 5 lesions was 0.87 (95% confidence interval: 0.77, 094).

The ICCs and results of Bland-Altman analysis for other VERDICT-derived metrics are provided in Table E4 and Figure E1 (online), respectively.

### Image Quality Assessment

No difference was found in overall image quality between ADC and FIC maps (mean score, 3.1 vs 3.3, respectively; adjusted *P* = .90). The interobserver weighted κ of image quality scores was 0.23 for FIC maps and 0.36 for ADC maps. The differences between mean ADC and FIC contrast-to-noise ratio were not statistically significant (1.84 and 1.74, respectively; *P* > .99).

The results of image quality assessment for other VERDICT metrics are provided in Table E5 and Figure E2 (online).

### Gleason Grade Differentiation

The FIC and ADC values for each Gleason grade group, along with other VERDICT-derived parameters, are provided in Table E6 (online).

**Figure 3:**
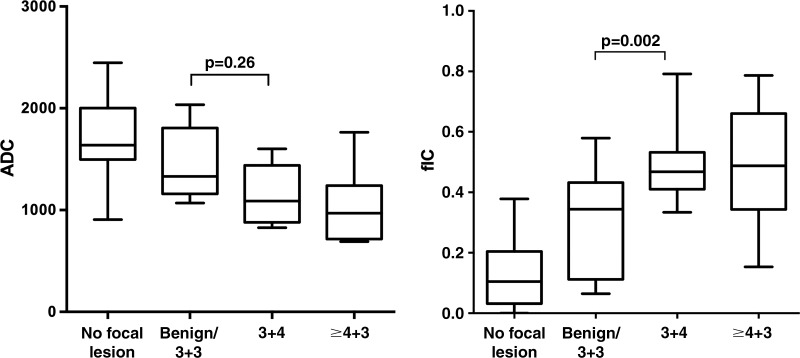
Box-and-whisker plots show distribution of, left, apparent diffusion coefficient (ADC) (in square millimeters per second) and, right, Vascular, Extracellular, and Restricted Diffusion for Cytometry in Tumors (VERDICT) MRI–determined intracellular volume fraction (*fIC*) (in fraction of signal, where 1.0 = total signal). Key differences in metrics between benign and/or Gleason grade 3+3 lesions and Gleason grade 3+4 lesions are shown, whereby *P* = .26 for ADC and *P* = .002 for intracellular volume fraction. Corrected *P* values for ADC after Bonferroni correction were as follows: no focal lesion versus benign and/or Gleason grade 3+3 lesions, *P* = .011; no focal lesion versus Gleason grade 3+4 lesion, *P* ≤ .001; no focal lesion versus lesions with Gleason grade 4+3 or higher, *P* ≤ .001; benign and/or Gleason 3+3 lesions versus focal lesions with Gleason grade 3+4, *P* = .26; benign and/or Gleason grade 3+3 lesions versus focal lesions with Gleason grade of 4+3 or higher, *P* = .047; and Gleason grade 3+4 lesions versus focal lesions with Gleason grade of 4+3 or higher, *P* > .99. Corrected *P* values for intracellular volume fraction after Bonferroni correction were as follows: no focal lesion versus benign and/or Gleason grade 3+3 lesions, *P* ≤ .001; no focal lesion versus Gleason grade 3+4 lesions, *P* ≤ .001; no focal lesion versus focal lesions with Gleason grade of 4+3 or higher, *P* ≤ .001; benign and/or Gleason grade 3+3 lesions versus focal lesions with Gleason grade 3+4, *P* = .002; benign and/or Gleason grade 3+3 lesions versus focal lesions with Gleason grade of 4+3 or higher, *P* = .006; and Gleason grade 3+4 lesions versus focal lesions with Gleason grade of 4+3 or higher, *P* > .99.

**Figure 4:**
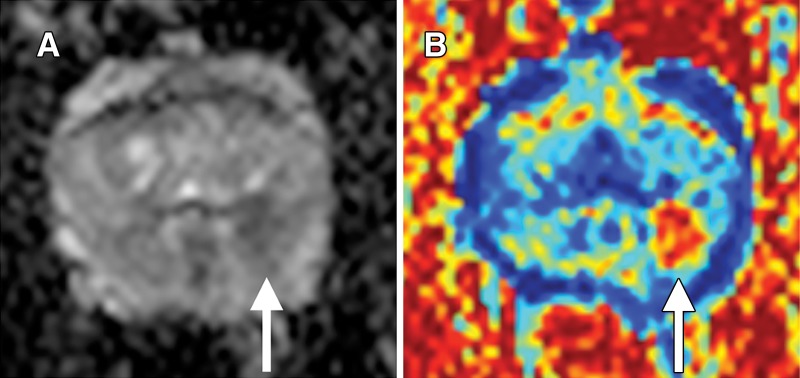
Images in a 57-year-old man with targeted biopsy–proven Gleason 3+4 prostate cancer. *A*, Apparent diffusion coefficient (ADC) map shows reduced ADC in left peripheral zone at 3 to 5 o’clock (arrow). *B*, Vascular, Extracellular, and Restricted Diffusion for Cytometry in Tumors (VERDICT) intracellular volume fraction map. Tumor (arrow) is very conspicuous.

**Figure 5:**
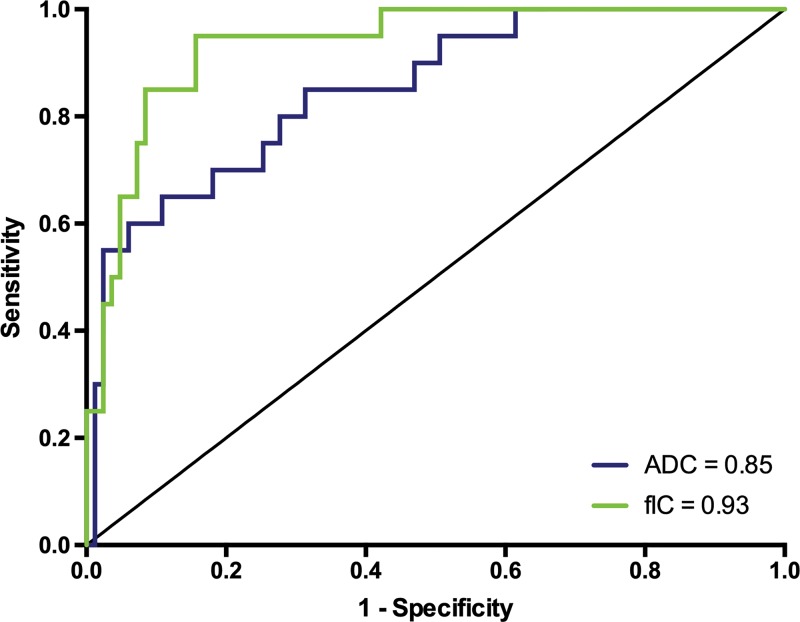
Receiver operating characteristic curves and corresponding area under the curve values for apparent diffusion coefficient (ADC) and intracellular volume fraction measurement (*fIC*) obtained with Vascular, Extracellular, and Restricted Diffusion for Cytometry in Tumors MRI to differentiate benign and/or Gleason grade 3+3 prostate lesions from lesions with Gleason grade of 3+4 or 4+3 or higher.

The distribution of ADC and FIC according to Gleason grade group is shown in Figure 3. The mean FIC for Gleason grade 3+4 lesions was higher than that for benign and/or Gleason grade 3+3 lesions (mean FIC, 0.49 vs 0.31, respectively; *P* = .002). The mean ADC for Gleason grade 3+4 lesions was similar to that for benign and/or Gleason grade 3+3 lesions (mean ADC, 1.42 vs 1.16 × 10^-3^ mm^2^/sec, respectively; *P* = .26). An example case demonstrating Gleason grade 3+4 disease on FIC and ADC maps is shown in Figure 4. The diagnostic performance of ADC and FIC for differentiating benign or Gleason grade 3+3 lesions from lesions with a Gleason grade of 3+4 or 4+3 or higher was good (area under the receiver operating characteristic curve: 0.85 [95% confidence interval: 0.76, 0.94] and 0.93 [95% confidence interval: 0.88, 0.99], respectively; *P* = .22) (Fig 5).

The performance of other VERDICT MRI–derived metrics in the differentiation of Gleason grade is provided in Figures E3 and E4 (online). Extravascular extracellular volume fraction and vascular volume fraction did not show statistically significant differences between Gleason grade 3+3 and Gleason grade 3+4 groups. Furthermore, the areas under the receiver operating characteristic curve for differentiating between benign or Gleason grade 3+3 lesions and Gleason grade 3+4 or 4+3 or higher lesions were lower than for ADC.

## Discussion

Signal on diffusion MRI is derived from contributions from intracellular water, water in the extracellular extravascular space, and water in the microvasculature. Because the fraction of each of these compartments differs between each Gleason grade (3), we hypothesized that Vascular, Extracellular, and Restricted Diffusion for Cytometry in Tumor (VERDICT) MRI–derived metrics may provide higher biologic specificity than the apparent diffusion coefficient as a marker of prostate cancer aggressiveness. Our results showed that fractional intracellular volume was greater for Gleason grade 3+4 lesions compared with benign and/or Gleason grade 3+3 lesions (mean, 0.49 vs 0.31, respectively; *P* = .002). For the same comparisons, the apparent diffusion coefficient showed no difference between groups (mean, 1.42 vs 1.16 × 10^-3^ mm^2^/sec; *P* = .26). Furthermore, we found that the diagnostic performance of fractional intracellular volume was comparable to that of the apparent diffusion coefficient (area under the receiver operating characteristic curve, 0.93 vs 0.85, respectively) for differentiating between the two groups. This suggests that the fractional intracellular volume has an equivalent or potentially greater improved performance in the differentiation between disease states.

Tumors with Gleason grade 4 have distinct genomic signatures (15), greater metastatic potential (16), and unfavorable survival outcomes (17). Metrics that can help classify cancers containing Gleason 4 have multiple potential clinical applications, including the noninvasive monitoring of patients with prostate cancer on active surveillance, more accurately avoiding and/or triggering biopsies, and as part of risk-stratification algorithms for guiding treatment decisions.

To be clinically useful, quantitative metrics must demonstrate good repeatability (18). FIC achieved high levels of repeatability (ICC ≥ 0.87) comparable to previously reported levels of ADC repeatability (19,20) and favorable to other diffusion models in prostate cancer. For example, one group (21) compared the repeatability of ADC with parameter estimates from stretched exponential, diffusion kurtosis, and biexponential models in the human prostate and found that although monoexponential fits and diffusion kurtosis achieved ICCs of approximately 0.75, stretched exponential and biexponential parameters achieved ICCs of approximately 0.25. Another group of investigators showed that the ICCs of pseudodiffusion coefficient and perfusion fraction from intravoxel incoherent motion and α from a stretched exponential model were 0.25, 0.42, and 0.64, respectively, even when calculated from two sets of identical *b* values in a single acquisition (22).

Complex imaging techniques often suffer from poor image quality when applied more widely, yet in our cohort we found no significant difference in qualitative or quantitative image quality measures between FIC and ADC maps. This could be expected as both techniques use the same echo-planar imaging readout to generate images. To our knowledge, two studies have evaluated image quality in prostate multiparametric MRI and used a similar five-point scale to assess the overall image quality of ADC images (11,12). These studies showed a mean image quality score of 3.18 and 3.03, respectively; thus, the quality of our ADC and FIC images (mean image quality score, 3.06 and 3.30, respectively) was comparable to those found in the literature.

The main limitation of our study is the number of participants and time constraints which precluded performing ADC repeatability as a comparator. However, similar studies have had eight or fewer study participants (23), which emphasizes that interval repeatability examinations are difficult to perform given participant tolerance issues and time limitations of clinical workflows (where we were allocated a 1-hour imaging slot). Small sample size likely limited our ability to examine differences in diagnostic performance (assessed with comparison of areas under the receiver operating characteristic curve) for FIC and ADC values.

In summary, intracellular volume fraction determined with Vascular, Extracellular, and Restricted Diffusion for Cytometry in Tumor (VERDICT) MRI enables better differentiation of Gleason 4 lesions from benign and/or Gleason 3+3 lesions in prostate cancer with a high level of repeatability and an image quality similar to that of apparent diffusion coefficient values. Reproducibility and multicenter clinical evaluation remain the next steps in VERDICT development (18).

## APPENDIX

Appendix E1, Tables E1–E6 (PDF)

## SUPPLEMENTAL FIGURES

Figure E1:

Figure E2:

Figure E3:

Figure E4:
