# Supplementary material for: VERDICT MRI for Prostate Cancer: Intracellular Volume Fraction versus Apparent Diffusion Coefficient
Source: Radiology. 2019 Apr 2;291(2):391–7. doi: 10.1148/radiol.2019181749 (PMC6493214; doi:10.1148/radiol.2019181749)
Supplement: Appendix E1, Tables E1–E6 (PDF) [file ry181749suppa1.pdf]

©RSNA, 2019
10.1148/radiol.2019181749

# Appendix E1

## Introduction

In this section, we present further results regarding two other Vascular, Extracellular, and Restricted Diffusion for Cytometry in Tumors (VERDICT) MRI parameters, namely the extravascular, extracellular volume fraction (FEES) and vascular volume fraction (FVASC) metrics. We also provide more detailed information regarding analytical methods and the distribution of ADC and all VERDICT metrics in each Gleason grade group.

## Methods

Acquisition parameters for the standard multiparametric (mp) prostate MRI and VERDICT MRI are provided in Tables E1 and E2. Gadoteridol (Prohance, from Bracco, Milan, Italy) was used for contrast-enhanced imaging.

## Analytical Methods

FEES and FVASC metrics were extracted using the same regions of interest (ROIs) as for the intracellular volume fraction (FIC) maps, whereby the VERDICT parameter estimates were extracted by fitting the VERDICT model to the data, according to reference 2.

The scale used by the two independent board-certified radiologists to provide an assessment of overall image quality is provided in Table E3.

## Results

Further results concerning the repeatability (Table E4 and Fig E1), overall image quality (Table E5), contrast-to-noise ratio (CNR, Fig E2) and Gleason grade differentiation (Figs E3 and E4) for FEES and FVASC are provided. For the qualitative assessment of overall image quality, the interobserver weighted κ was 0.31 for FEES and 0.42 for FVASC.

Finally, the distribution of ADC and VERDICT metrics in each Gleason grade group is presented in Table E6.

**Table E1: Acquisition parameters for clinical multiparametric (mp) MRI at 3T.**

| Sequence | TR | TE | BW | FoV | ST | Gap | TSE factor | PD | FS | ACQ | Total scan duration |
|---|---|---|---|---|---|---|---|---|---|---|---|
| T2 sag REF | 1579 | 100 | 217.3 | 240 | 5 | 5 | 20 | A > P | No | 120 × 89 | 00:18.9 |
| T2 TSE coronal | 6128 | 100 | 160.7 | 180 | 3 | 3 | 16 | R > L | No | 300 × 290 | 05:55.4 |
| T2 TSE axial | 5407 | 100 | 160.7 | 180 | 3 | 0 | 16 | R > L | No | 300 × 290 | 05:13.6 |
| DWI 0 150 500 1000 | 2753 | 80 | 10.8 | 220 | 5 | 0 | — | A > P | SPAIR | 168 × 169 | 05:16.5 |
| DWI b2000 | 2000 | 78 | 9.9 | 220 | 5 | 0 | — | A > P | SPIR | 168 × 169 | 03:40.0 |
| DCE 20 dyn mod SENSE | 5.8 | 2.8 | 246.1 | 180 | 3 | 0 | — | R > L | SPAIR | 140 × 162 | 04:14.1 |

Note.–TSE; turbo spin echo, DWI; diffusion-weighted imaging, DCE; dynamic contrast enhanced, SENSE; sensitivity encoding for fast MRI, TR; Repetition time, ms TE; Echo time, ms, BW; Bandwidth Hz/pixel, FoV; field of view, mm, ST; Slice thickness, mm, Gap, mm, PD; Phase encoding direction, FS; fat saturation technique, ACQ; acquisition matrix, mm.

## Table E2: Diffusion gradient parameters for Vascular, Extracellular and Restricted Diffusion for Cytometry in Tumors (VERDICT) MRI.

| b value, s/mm$^2$ | Δ/δ, ms | TE, ms | TR, ms | \|G\|, T/m | NEX |
|---|---|---|---|---|---|
| 90 | 23.8/3.9 | 50 | 2482 | 0.061 | 4 |
| 500 | 31.3/11.4 | 65 | 2482 | 0.044 | 6 |
| 1500 | 43.8/23.9 | 90 | 2482 | 0.032 | 6 |
| 2000 | 34.3/14.4 | 71 | 3945 | 0.068 | 6 |
| 3000 | 38.8/18.9 | 80 | 3945 | 0.060 | 6 |

Δ= timing between gradient pulses, δ= gradient pulse duration, |G|= gradient strength, NEX = number of excitations, T = Tesla, TE = echo time, TR = repetition time.

## Table E3: Overall image quality score scale

| | |
|---|---|
| 1 | Very poor quality, considered nondiagnostic (artifacts on all slices, scans uninterpretable). |
| 2 | Poor quality with some impairment of diagnostic quality (substantial artifacts, but still interpretable) |
| 3 | Satisfactory quality without impairment of diagnostic quality (some artifacts present) |
| 4 | Good quality (hardly any artifacts) |
| 5 | Excellent quality (no artifacts present) |

## Table E4: Intraclass Correlation Coefficients (ICCs) of Vascular, Extracellular and Restricted Diffusion for Cytometry in Tumors (VERDICT) parameters for the repeatability cohort (*n* = 42)

| Parameter | No focal lesion TZ | No focal lesion PZ | Focal lesions (*n* = 34) |
|---|---|---|---|
| FEES | 0.88 (0.78–0.94) | 0.88 (0.67–0.90) | 0.77 (0.54–0.89) |
| FVASC | 0.80 (0.65–0.89) | 0.80 (0.65–0.90) | 0.76 (0.55–0.89) |

Note.-95% confidence intervals (CI) are shown in parentheses. FEES = extravascular, extracellular volume fraction, FVASC = vascular volume fraction. ICC = intraclass correlation coefficient, TZ = transition zone, PZ = peripheral zone.

## Table E5: Results of image quality assessment of the biopsy cohort (42 participants) for both readers (*n* = 84)

| | ADC | FIC | FEES | FVASC |
|---|---|---|---|---|
| 1: Very poor | 7 | 0 | 4 | 10 |
| 2: Poor | 14 | 17 | 19 | 34 |
| 3: Fair | 36 | 34 | 42 | 32 |
| 4: Good | 21 | 24 | 14 | 8 |
| 5: Excellent | 6 | 9 | 5 | 84 |
| Mean | 3.06 | 3.30 | 2.96 | 2.45 |

Note.-results are number of scans for each VERDICT map scoring each level of image quality. ADC; apparent diffusion coefficient, FIC; intracellular volume fraction, FEES; extravascular, extracellular volume fraction, FVASC; vascular volume fraction. Corrected *P* values following Dunn's corrected ANOVA were as follows: ADC versus FIC *P* = .90, ADC versus FEES *P* > .99, ADC versus FVASC *P* = .0001, FIC versus FEES *P* = .41 FIC versus FVASC *P* < .0001, FEES versus FVASC *P* = .0006.

**Table E6: Distribution of Apparent Diffusion Coefficient (ADC) and Vascular, Extracellular and Restricted Diffusion for Cytometry in Tumors (VERDICT) parameters in each Gleason grade group.**

| Parameter | No focal lesion | Focal lesion: benign/3+3 | Focal lesion: 3+4 | Focal lesion: ≥ 4+3 |
|---|---|---|---|---|
| ADC | 1.71 ± 0.35 | 1.42 ± 0.32 | 1.16 ± 0.30 | 1.03 ± 0.36 |
| FIC | 0.12 ± 0.11 | 0.31 ± 0.17 | 0.49 ± 0.12 | 0.49 ± 0.20 |
| FEES | 0.57 ± 0.14 | 0.47 ± 0.13 | 0.40 ± 0.13 | 0.30 ± 0.15 |
| FVASC | 0.30 ± 0.11 | 0.21 ± 0.12 | 0.12 ± 0.09 | 0.19 ± 0.08 |

Note.-Parameters are expressed as mean ± SD. ADC = apparent diffusion coefficient (x $10^{-3}$ $mm^2/s$) FIC = intracellular volume fraction, FEES = extravascular, extracellular volume fraction, FVASC = vascular volume fraction. All VERDICT parameters are expressed as fractions, whereby the FIC + FEES + FVASC = 1.
