# Supplementary figures and images for: VERDICT MRI for Prostate Cancer: Intracellular Volume Fraction versus Apparent Diffusion Coefficient
Source: Radiology. 2019 Apr 2;291(2):391–7. doi: 10.1148/radiol.2019181749 (PMC6493214; doi:10.1148/radiol.2019181749)

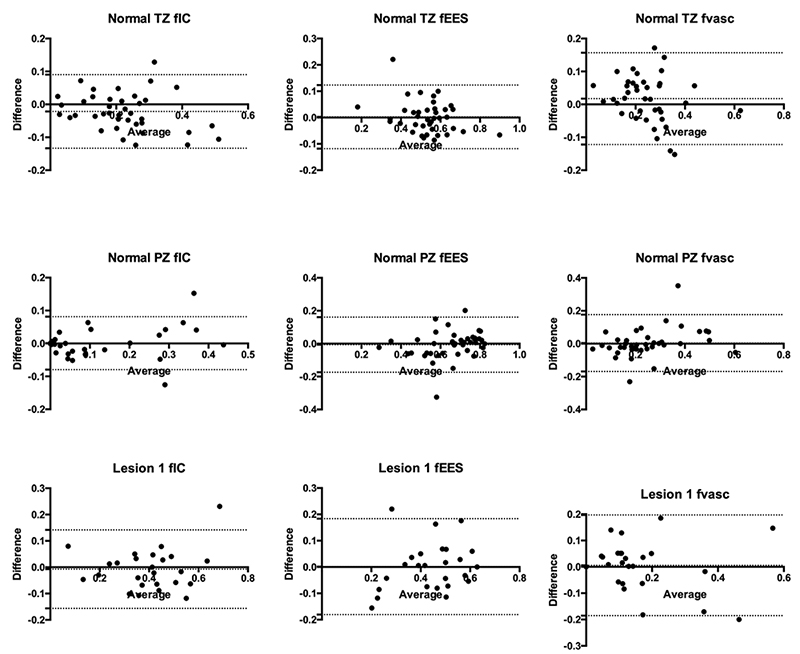

Supplement: Figure E1: [file ry181749suppf1.jpg]

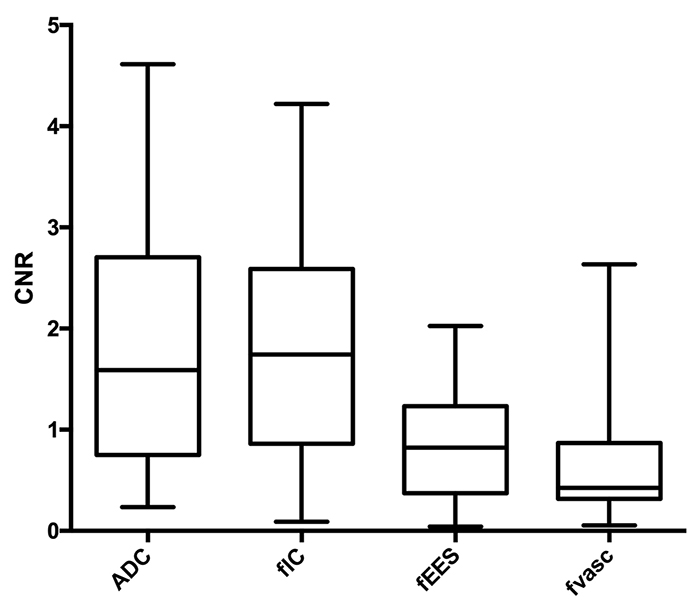

Supplement: Figure E2: [file ry181749suppf2.jpg]

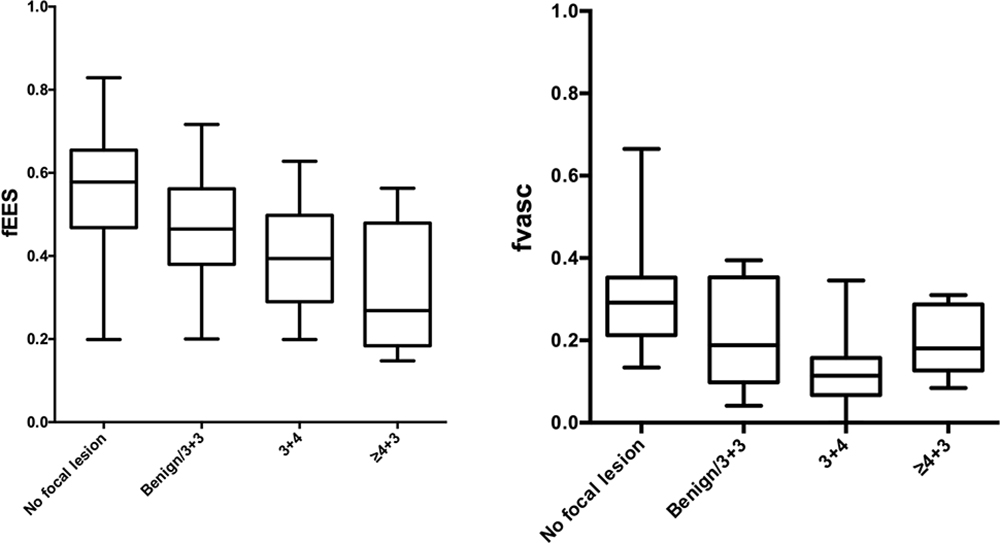

Supplement: Figure E3: [file ry181749suppf3.jpg]

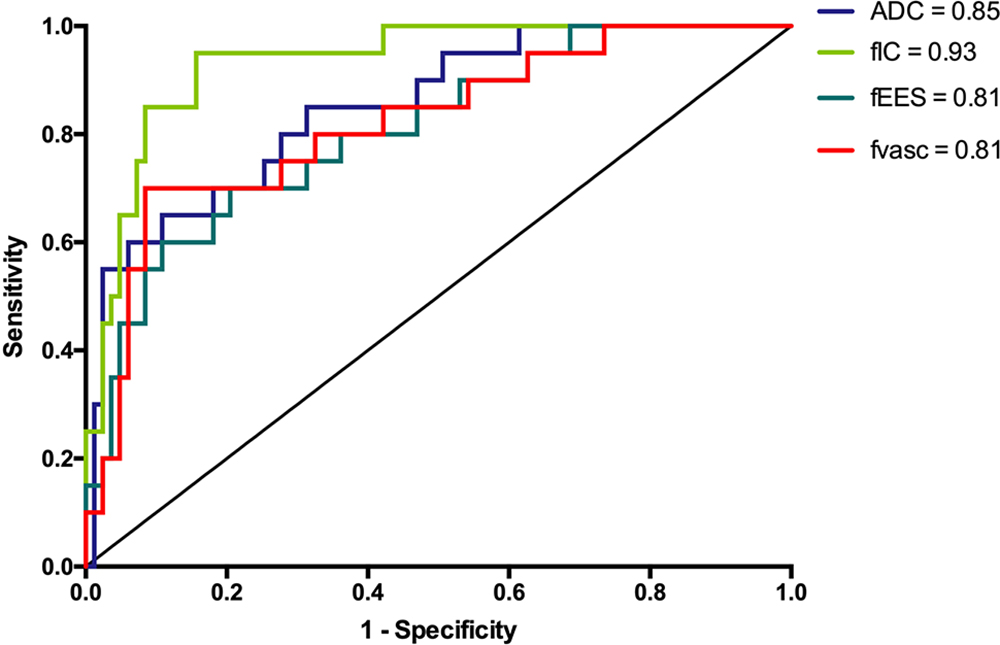

Supplement: Figure E4: [file ry181749suppf4.jpg]
